# Diagnostic values of 2 different techniques for controversial lumbar disc herniation by conventional imaging examination: 3D-DESS vs. CT plain scan

**DOI:** 10.3389/fphys.2022.953423

**Published:** 2022-09-14

**Authors:** Wei Liu, Jinhua Chen, Yanan Zhang, Xu Wang, Junwen Zheng, Aibing Huang, Chunmao Chen, Jian Bian, Lei Yang, Haijun Li

**Affiliations:** ^1^ Department of Rehabilitation, Taizhou People’s Hospital, Nanjing Medical University, Taizhou, China; ^2^ Department of Diagnostic Radiology, Taizhou People’s Hospital, Nanjing Medical University, Taizhou, China; ^3^ Graduate School, Dalian Medical University, Dalian, China; ^4^ Department of Orthopedics, Taizhou People’s Hospital, Nanjing Medical University, Taizhou, China; ^5^ Department of Orthopedics, Postgraduate Training Base of Dalian Medical University (Taizhou People’s Hospital), Taizhou, China

**Keywords:** lumbar disc herniation, spine, MRI, multidetector computed tomography, three-dimensional double-echo steady-state

## Abstract

**Background:** The aim of this study was to explore the significance of three-dimensional double-echo steady-state (3D-DESS) sequence and multidetector computed tomography (CT) plain scan in the diagnosis of lumbar disc herniation (LDH) remaining controversial in conventional magnetic resonance imaging (MRI), and to compare the efficiency between 3D-DESS and CT in diagnosing controversial patients by conventional MRI.

**Methods:** A total of 61 patients with controversial LDH diagnosed by conventional MRI were collected. Before operation, the disease of these patients was further confirmed by 3D-DESS sequences and continuous CT plain scan from L3 to S1. Finally, for patients whose postoperative curative effect was marked and symptoms were obviously alleviated, the sensitivity, specificity and accuracy.

**Results:** Among, 59 patients with remarkably relieved symptoms after operation were included, and 2 patients with varying degrees of non-remission of pain and partial dysfunction after operation were excluded. The sensitivity, specificity and accuracy of 3D-DESS were 94.6, 100 and 94.9%, respectively, and those of CT were 75.0, 33.3 and 72.9%, respectively.

**Conclusion:** 3D-DESS is a very useful diagnostic method for patients with some special types of LDH that remain controversial in conventional imaging diagnostic methods. Through 3D-DESS, the morphology of lumbosacral nerve roots can be directly observed, which is conducive to the improvement of the sensitivity, specificity and accuracy, thus further reducing the misdiagnosis rate. Moreover, 3D-DESS plays a guiding role in the formulation of operative methods.

## Introduction

Lumbar disc herniation (LDH) has become the leading cause of low back pain (LBP) and sciatica in recent years (Konstantinou and Dunn, 2008; Martin et al., 2008; Hoy et al., 2010; Jacobs et al., 2011), and minimally invasive surgeries have increasingly become the best surgical method for LDH, among which the dominating percutaneous transforaminal endoscopic discectomy (PTED) has witnessed great success in the treatment of the disease. It removes the herniated nucleus pulposus and hyperplastic osteophyte through the “safety triangle” of the intervertebral foramen, thereby relieving the compression to nerve roots. However, the imaging results consistent with the symptoms and signs of patients are the prerequisite for surgical treatment. The establishment of the channel represents that the probe is restrained around the channel (Hoogland et al., 2006; Lee et al., 2010; Choi and Park, 2016). Hence, the PTED requires single accurate puncture to the target location, and the strict requirement for locating the disc herniation before surgery not only contributes to accurate diagnosis, but also provides guidance for the precise planning of surgical operation.

Magnetic resonance imaging (MRI), the most frequently used examination method to evaluate patients with sciatica, exhibits a diagnostic accuracy of 97% and high reliability among observers (Kreiner et al., 2014). For patients whose medical history, symptoms and signs are consistent with MRI results, MRI is recommended as a non-invasive examination suitable for the diagnosis of LDH. Nevertheless, it often happens that the clinical symptoms and signs of patients are inconsistent with the results of MRI examination, and the area near the intervertebral foramen is prone to missed diagnosis by cross-sectional and sagittal MR images (Baba et al., 1997). The oblique coronal MRI of the lumbar can display the nerve roots inside and outside the intervertebral foramen, but it is not obviously beneficial for LDH patients with spinal deformity (Lee et al., 2009). Owing to the complex anatomical structure of lumbosacral vertebra, conventional MRI plain scan can only show the nerve roots at the cross-sectional level. Besides, different slice thickness of scanning and the interference of other artifact signals make it difficult for clinicians to observe the shape of nerve roots intuitively and completely, thus leading to the inconsistency of MRI diagnosis results with clinical symptoms.

With the development of cross-sectional imaging and computed tomography (CT), including multidetector CT (CT), the diagnostic efficiency of CT is close to that of MRI, so CT can be used as a substitute for MRI (Notohamiprodjo et al., 2017). However, when the protrusion inside or outside the intervertebral foramen has a roughly equal density to adjacent nerve roots and posterior root ganglia, it may bring some difficulties to diagnosis and even lead to misdiagnosis as tumors (Schlesinger et al., 1992). Additionally, the discontinuous CT scan of the lower layer of vertebral pedicles may also lead to missed diagnosis. Hence, thin-slice scanning of the upper and lower vertebral pedicles should be employed to avoid missed diagnosis, and coronal reconstruction should be performed if necessary (Jackson and Glah, 1987).

Three-dimensional double-echo steady-state (3D-DESS) is an MRI method that utilizes 3D gradient-echo sequences to achieve thin-slice scanning (Hardy et al., 1996; Eckstein et al., 2006; Moriya et al., 2009). On the lumbosacral plexus, 3D-DESS can clearly exhibit the changes of adjacent intervertebral discs and the compression of dural sacs and spinal cords, and almost all intervertebral discs with morphological changes are subjected to signal reduction. When the nerve roots are compressed by the herniated nucleus pulposus, such phenomena as nerve root indentation, ganglion strengthening and nerve root swelling or interruption may appear. Observers can see the lesions of the responsible segment more directly than those in conventional cross-sectional and sagittal MRI.

In this study, with findings during operation as reference standards, samples were retrospectively included to explore the significance of 3D-DESS imaging of lumbosacral nerve root in the diagnosis of controversial LDH patients undergoing conventional imaging examination, and to compare the difference in efficiency between 3D-DESS sequences and CT plain scan in diagnosing controversial patients. Besides, the application of 3D-DESS technology was expected to improve the diagnosis rate of patients with special types of LDH or controversial LDH, reduce the misdiagnosis rate, and finally guide the decision-making of operative methods.

## Patients and methods

### Patient selection

This study was approved by the Ethics Committee of Taizhou People’s Hospital (Ethical Approval No.: KY202009201). A total of 271 patients with typical symptoms of sciatica admitted to the hospital from January 2015 to October 2019 were retrospectively included. These patients could not be definitely diagnosed by MRI, and they underwent preoperative 3D-DESS and CT scan. Next, the diagnostic efficiency of the obtained images was evaluated with the intraoperative findings as the gold standards. Demographic information for all the enrolled individuals was shown in Table 1.

**TABLE 1:** Basic data of patients.

Item	n
Age (years old)	61.0 ± 1.4
Male	28
Female	31
Segment	
L3/L4	4
L4/L5	29
L5/S1	26
Patients receiving surgery	59

For patients with typical sciatica symptoms, conventional MRI examination was performed at first. However, for patients who could not be diagnosed by conventional MRI, including those without LDH and those with LDH not conforming to clinical symptoms and signs, 3D-DESS examination and CT scan (scanning segment: L3-S1, slice thickness of scanning: 1 mm) were continued to further confirm the diagnosis. Then the patients definitely diagnosed through either of the two methods, who had consistent LDH location as well as clinical symptoms and signs, were treated by operation, and the correlations of the size and location of LDH with nerve roots were recorded in detail.

The improvement of clinical symptoms before and after operation was assessed by the Visual Analogue Scale (VAS) scores (0 points: painless, and 10 points: the most painful). The Japanese Orthopaedic Association (JOA) score for low back pain (LBP) was adopted for preoperative and follow-up clinical evaluation. The recovery rate of JOA score was calculated using the formula as follows: recovery rate of JOA score = (follow-up score - preoperative JOA score)/(29 - preoperative JOA score) × 100% (Fujiwara et al., 2003). Later, in comparison with the preoperative condition, the improvement rate of VAS score was calculated using the following formula: improvement rate of VAS score = (preoperative VAS score - follow-up score)/preoperative VAS score × 100%. The patients were followed up by doctors through telephone inquiry and outpatient examination at 1 month and 3 months after operation.

Patients who meet the following criteria were enrolled in this study to evaluate the diagnostic efficiency of imaging examinations: 1) patients with typical LBP accompanied with radiation pain of lower limbs, including those with positive results in the straight leg raising test and femoral nerve traction test, 2) those with unsatisfactory outcomes accompanied with unbearable pain or dysfunction after 6 weeks of conservative treatment, 3) those with controversial diagnosis result of MRI plain scan of the lumbar vertebra, 4) those undergoing 3D-DESS examination and CT scan at the same time before operation, and 5) those subjected to surgical treatments, including lumbar fusion surgery and PTED, with a recovery rate of JOA score more than 45% and an improvement rate of VAS score more than 60% in the follow-up at 3 months after operation, which confirmed the operative effect.

Exclusion criteria were set as follows: 1) patients receiving no surgical treatment, or those who lacked detailed LDH information during operation, 2) those with incomplete imaging data, including MRI scan, 3D-DESS examination and CT scan, 3) those who were treated with lumbar surgery previously, 4) those who could not receive examination by MRI machine, had allergic reaction to contrast medium or had contraindications to MRI examination, or 5) those with a recovery rate of JOA score less than 45% or an improvement rate of VAS score less than 60% in the follow-up at 3 months after operation.

In this study, all CT examinations were performed with Siemens 64-slice CT scanner (Siemens Medical Solutions, Erlangen, Germany). Besides, L3-S1 vertebral segments underwent continuous CT plain scan, during which the scanning plane was parallel to the transverse plane of the human body, with an interval of 1 mm.

In this study, 3-Tesla MRI machine (Skyra 3.0 T, Siemens AG, Erlangen, Germany) was utilized in conventional MRI examination and 3D-DESS sequences. The imaging data shown by conventional MRI examination included cross-sectional images of the L2 to S1 intervertebral disc planes parallel to the superior vertebral endplate (four segments in total, namely, L2/L3, L3/L4, L4/L5, L5/S1, providing three T2-weighted tomographic images for each intervertebral plane) and 7 T2-weighted sagittal images from L2 to S1, with the scanning covering the spinal canal.

The related scanning parameters of 3D-DESS sequences were shown in Table 2. To obtain images of all lumbar dorsal root ganglia (DRG) and spinal nerves, the imaging plane was set parallel to the longitudinal axis of the lumbar spinal canal, and the scanning was performed with L3 vertebra as the center. Maximum intensity projection (MIP) and multiplanar reconstruction (MPR) were utilized to obtain high-resolution images of the lumbosacral nerve roots. Moreover, an Aquarius 3D workstation equipped with automatic analysis 3D rendering software for commercial use (TeraRecon Inc, San Mateo, CA) was adopted for image processing.

**TABLE 2:** Imaging parameters of 3D-DESS sequences.

Imaging acquisition parameters	Values
Repetition time (ms)/echo time (ms)	14.60/6.00
Flip angle (degree)	25
FOV read (mm)/FOV phase (%)	328/81.3
Slice thickness (mm)	0.7
In-plane resolution (mm)	320*288
Slice gap (mm)	0.14
Pixel Bandwidth (Hz/pixel)	252
Acquisition time (min:s)	4:08

### Image evaluation

Images were analyzed using SkyView PACS V3.3.1.5 software (Yangtze River Ruiheng Software, Ltd., Nanjing, China), and the conclusion that preoperative MRI could not make a definite diagnosis was independently judged by two spinal surgeons in the same treatment group according to the clinical symptoms and signs of patients and the MRI data. The two spine surgeons had many years of experience in bone and spine examination, and possessed approximately the same proficiency in image interpretation, and were able to independently interpret the images. When the interpretation results were different, they made a unified decision after negotiation. If there were still controversies after negotiation, the interpretation results of the senior doctor would be regarded as the final results. CT and 3D-DESS image evaluation was also completed by the above two spinal surgeons, and all the evaluation details were recorded in the medical record data.

### Statistical analysis

All the image interpretation results of 3D-DESS and CT were compared with the intraoperative findings, and the sensitivity, specificity and accuracy of CT and 3D-DESS were calculated.

## Results

A total of 65 patients were definitely diagnosed by 3D-DESS examination (including 33 cases with herniated nucleus pulposus inside and outside the intervertebral foramen). Among them, 59 patients had obviously relieved symptoms after operation, so they were included in this study retrospectively, including 28 males and 21 females aged 28–41 years old, with an average age of 60.14 years old. The information of the patients included after screening was shown in Table 1. According to the surgeons’ findings during operation, there were 4 cases in L3/L4 segment, 29 cases in L4/L5 segment and 26 cases in L5/S1 segment. The sensitivity, specificity and accuracy of CT were 75.0, 33.3 and 72.9%. In addition, there were 42 cases with positive CT and 3D-DESS results, 0 cases with negative CT and 3D-DESS results, 12 cases with positive 3D-DESS results but negative CT results, and 5 cases with positive CT results but negative 3D-DESS results. The diagnostic results of the two imaging methods were displayed in Table 3. Additionally, the sensitivity, specificity and accuracy of 3D-DESS diagnosis were 94.6, 100 and 94.9%, respectively.

**TABLE 3:** Comparison of diagnostic efficiency of 3D-DESS and CT fore* Data were the numbers of patients.

Techniques	Predict	Intraoperative finding		Sensitivity (%)	Specificity (%)	Accuracy (%)
		True*	False*			
3D-DESS	Positive	53	0	94.6	100.0	94.9
	Negative	3	3			
CT	Positive	42	2	75.0	33.3	72.9
	Negative	14	1			

*Data were the numbers of patients.

## Discussion

**FIGURE 1:**
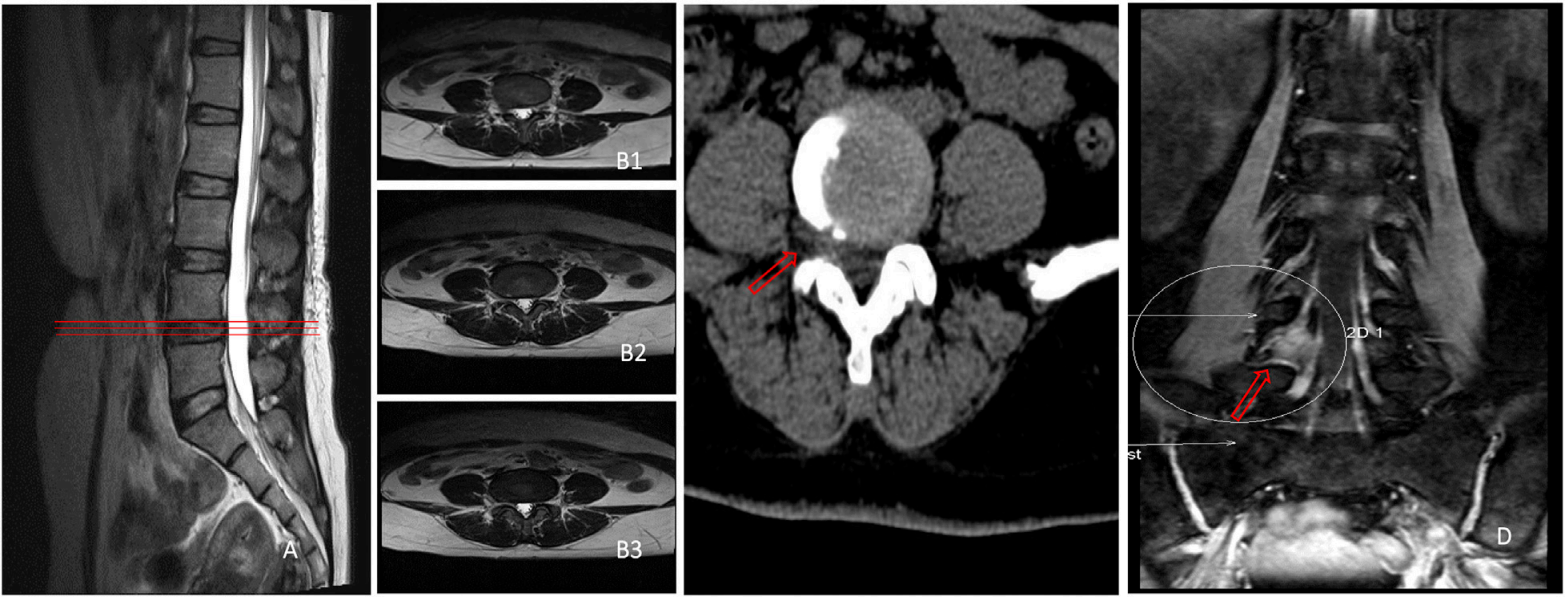
No suspicious disc herniation was found in L4/L5 segment in both sagittal **(A)** and cross-sectional MRI **(B1–B3)**, only one suspicious herniated nucleus pulposus existed on the extreme lateral side of L4/5 segment in cross-sectional CT **(C)**, and a huge nucleus pulposus could be seen to compress the right L4 nerve root under the armpit in 3D-DESS **(D)**.

Conventional MRI scan is a common diagnostic method for LDH, and central disc herniation and LDH can be easily diagnosed by conventional cross-sectional MR images (Kreiner et al., 2014). However, the imaging diagnosis results often turn out to be non-conforming to the typical clinical symptoms of patients. For example, as shown in Figure 1, the patient had typical symptoms of LBP with radiation pain in the right lower limb, and the clinical symptoms and signs all confirmed the compression of the L4 nerve root in the right lower limb. Besides, no remarkable abnormality was found in the L4/L5 segment in the cross-sectional and sagittal scans of conventional MRI, but through 3D-DESS, the huge free nucleus pulposus hidden under the armpit of the right L4 nerve root could be easily found. In a retrospective study on 271 patients with controversial results of conventional MRI, it was discovered through 3D-DESS and CT examinations that diagnosis results conforming to clinical symptoms could be obtained from 65 (24.0%) patients, and the symptoms of 59 patients (21.8%) were notably alleviated after operation since 3D-DESS and CT scan provide more information than conventional MRI. In the conventional MRI scanning modes, the cross-sectional MRI only scans for 3–4 times per segment only at the level of intervertebral disc and that parallel to the intervertebral disc. Moreover, the sagittal images are also limited to the spinal canal. Generally, the scanning covers seven slices, without a larger range of high-frequency continuous scanning of lesion segments. Therefore, this conventional scan has certain blind areas, while images obtained from 3D-DESS sequences and CT plain scan are more likely to manifest panoramic images compared with the local images of conventional MRI, so more information can be obtained through 3D-DESS sequences and CT plain scan than conventional scanning. Another important reason is that the 3D-DESS sequences boast an imaging feature of simultaneously displaying the intervertebral disc, nerve roots and the surrounding ligamentum flavum and pedicles. In particular, they can clearly show the relationship between the herniated intervertebral disc tissues and the shape of nerve roots. Besides, 3D-DESS is more intuitive and vivid than conventional MRI scan (Figure 1), and the former avoids the blind areas of the latter.

**FIGURE 2:**
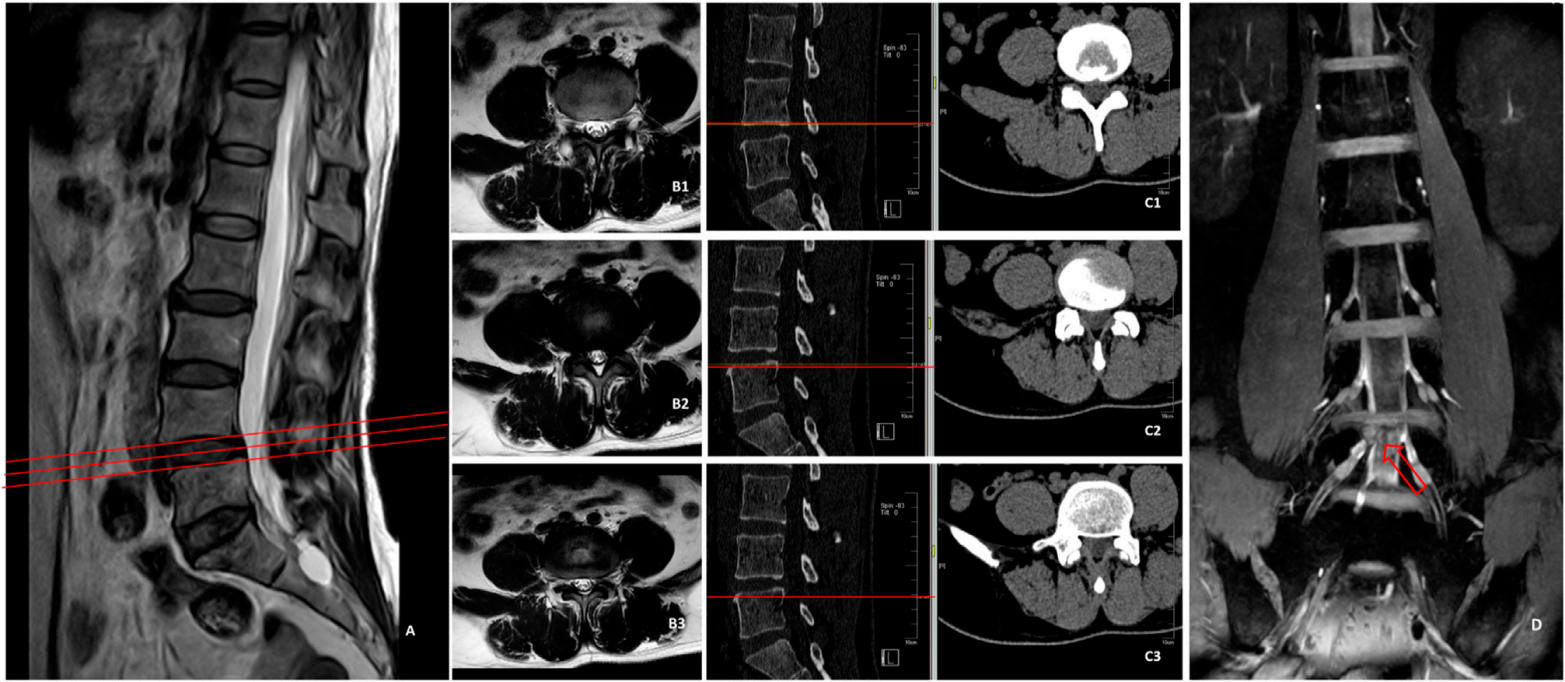
MRI **(A,B1–B3)** of L4/L5 segment showed no suspicious herniated intervertebral discs, CT **(C1–C3)** indicated that the right nucleus pulposus was slightly compressed, which was inconsistent with symptoms, while 3D-DESS image **(D)** indicated that the herniated disc tissue compressed L5 nerve root in the nerve root canal area.

It has been previously shown that the application of magnetic resonance neuroimaging (MRN) system can improve the diagnostic efficiency by 7% (Chhabra et al., 2016). After the nerve root is separated from the dural sac to the intervertebral foramen, the whole walk path is clearly displayed, and it is unmatched by other imaging methods in terms of clearly revealing the relationship between the herniated intervertebral disc and the nerve root. More information can be obtained through intensive CT scanning than conventional MRI, which effectively helps to avoid the blind areas of conventional MRI scan. Experienced physicians can generally make a definite diagnosis by carefully observing the images of cross-sectional CT scan slice by slice. Nonetheless, there are some exceptions. The herniation of intervertebral disc of some patients is very small, but it just compresses the nerve root, which is often ignored by CT scan. According to the imaging examination (Figure 2), MRI and CT only showed that the nucleus pulposus on the right side of L4/L5 segment was slightly compressed, which was inconsistent with symptoms. However, 3D-DESS images indicated that the herniated intervertebral disc tissue compressed the L5 nerve root in the nerve root canal area, indicating definite diagnosis.

**FIGURE 3:**
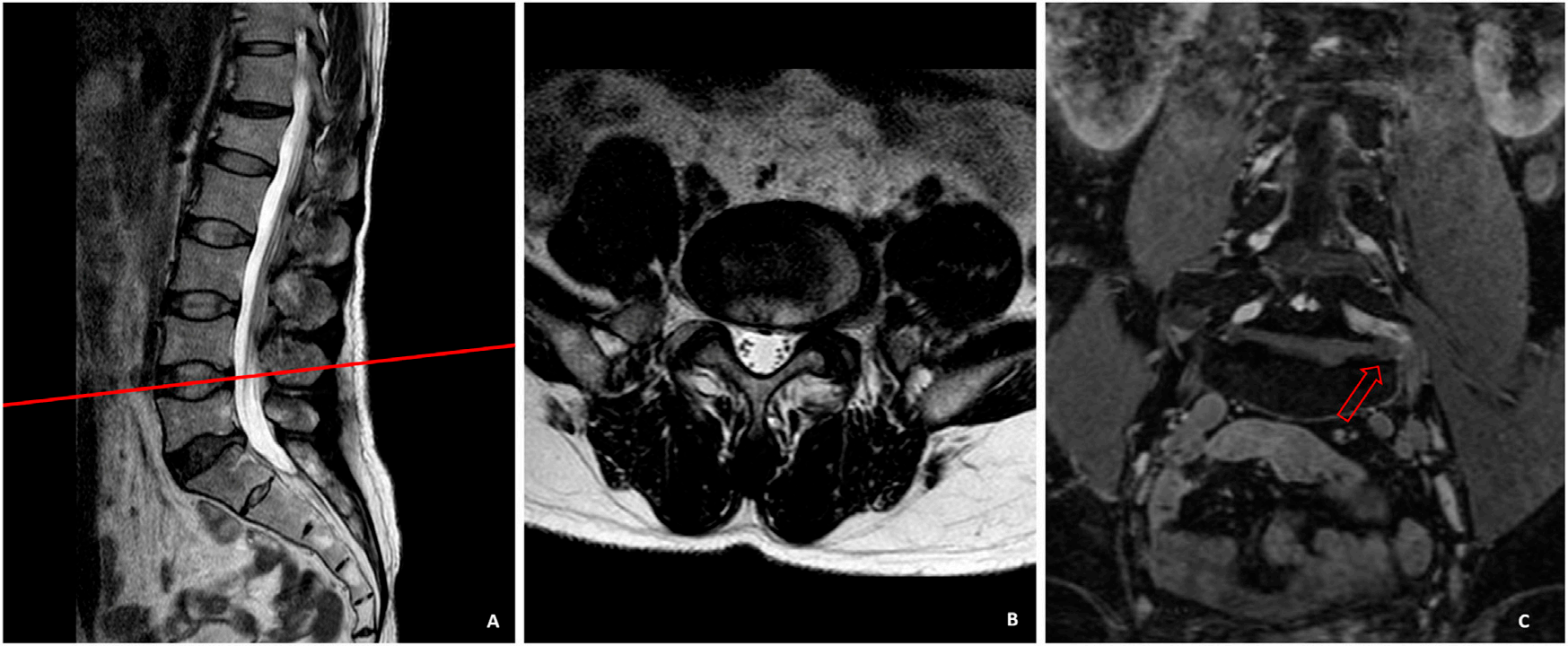
In sagittal **(A)** and cross-sectional **(B)** MRI, the herniated nucleus pulposus tissues near the intervertebral foramen were easily ignored. The compression of the nucleus pulposus of L4/L5 segment to the left L4 nerve root outside the intervertebral foramen could be intuitively observed on the 3D-DESS image **(C)**.

This study revealed that the LDH inside and outside the intervertebral foramen occupied a higher proportion in LDH patients that could not be diagnosed by conventional MRI scan. Previous studies have also shown that this type of herniation may be ignored using traditional conventional scanning techniques (Baba et al., 1997). In the present research, there were 35 cases of LDH inside and outside the intervertebral foramen, which were difficult to be diagnosed by conventional cross-sectional MR images. All of them were definitely diagnosed by 3D-DESS examination, and the symptoms of 33 patients were obviously alleviated after surgical treatment. Generally speaking, the focal eccentricity or asymmetry of disc contour and the change of nerve root in the area outside the intervertebral foramen are important imaging signs related to the diagnosis of LDH outside the intervertebral foramen (Moon et al., 2009). This method can definitely diagnose some patients, but as mentioned above, high-frequency continuous scanning in a larger range of lesion segments is generally not performed in conventional MRI. Hence, one of the important reasons for missed diagnosis is that there is a certain blind area in this conventional scan, especially when the nucleus pulposus inside or outside the intervertebral foramen is freed up. Besides, the tomographic scanning of conventional MRI parallel to the intervertebral disc plane often cannot cover the nucleus pulposus freed above the intervertebral disc plane and toward the pedicle, while the outlet root is often squeezed between the pedicle and the free nucleus pulposus. At that time, clinical patients suffer from unbearable LBP and lower limb radiation pain. As such, typical clinical symptoms of LDH appear in patients, which cannot be diagnosed by conventional MRI examination. As shown in Figure 3, the herniated nucleus pulposus tissues near the intervertebral foramen in the cross-sectional and sagittal MRI were easily ignored. However, the compression of the nucleus pulposus of L4/L5 segment to the left L4 nerve root outside the intervertebral foramen could be intuitively observed on 3D-DESS images. Additionally, the missed diagnosis of herniated intervertebral disc tissues in the medial and lateral areas of intervertebral foramen is also mainly caused by inadequate experience in this type of LDH of imaging doctors and spinal surgeons. Imaging doctors, in particular, pay more attention to disc herniation and nerve compression in the spinal canal area when they observe cross-sectional images, but often “turn a blind eye” to the compression outside the spinal canal. The intuitiveness and visualization of 3D-DESS make it easy to diagnose diseases, and can effectively avoid the missed diagnosis by imaging doctors. The advantage of CT plain scan lies in that it can obtain more information than conventional MRI through dense and continuous thin-slice scanning, especially the herniation of nucleus pulposus in the medial and lateral areas of intervertebral foramen. Through continuous thin-slice scanning, enough information can be obtained, and after careful slice-by-slice observation, the correct diagnosis can generally be realized. In contrast to 3D-DESS technology, it also requires doctors to be careful and experienced enough. Young spinal surgeons and imaging doctors with a lack of experience often make missed diagnosis. This can partly explain that the sensitivity, specificity and accuracy of 3D-DESS are higher than those of continuous CT scan.

**FIGURE 4:**
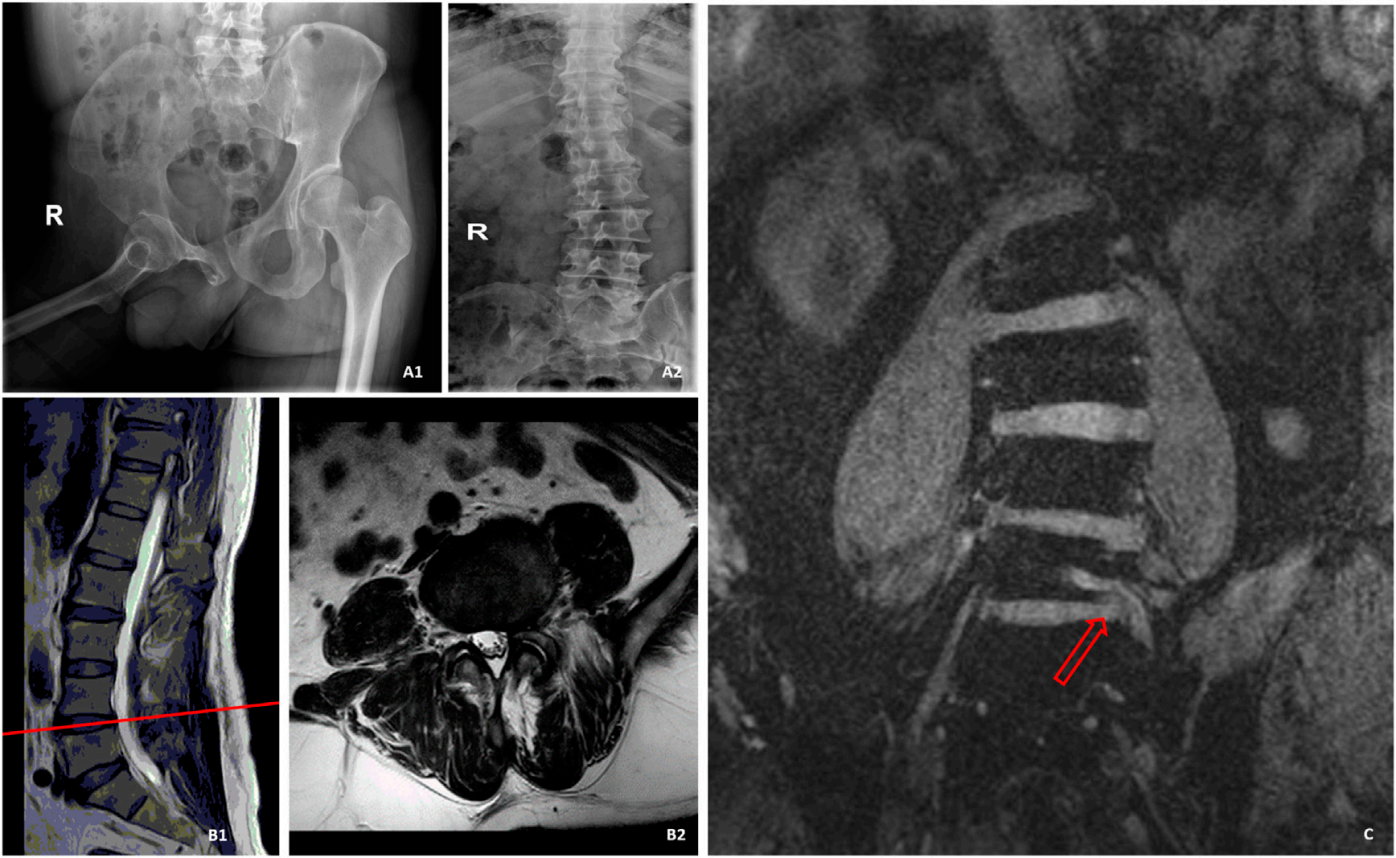
A patient with spinal deformity caused by poliomyelitis, who was admitted to hospital with LBP and radiation pain of left lower limb. **(A1)** was the pelvic X-ray of the patient. **(A2)** was the spine radiograph. B was the MR image of the L4/L5 segment of the spine, and no suspicious disc herniation was found. **(C)** was a 3D-DESS image. Owing to scoliosis, the left L5 nerve root could only be displayed on a single slice, and it could be observed that the left side of L5 nerve root was compressed by a huge nucleus pulposus.

3D-DESS is superior to CT and conventional MRI as it is not affected by the spinal deformity of patients. Patients with spinal deformity are challenging for conventional MRI and CT scan, since deformity brings difficulties to conventional scanning plan. The finally obtained images are related to the habits of the examination operators with no uniform standards, thereby often leading to the loss of important information related to diagnosis. On the other hand, it also poses challenges to the image observation after acquisition. Disc herniation in the spinal canal is often diagnosed according to whether the two sides are symmetrical or whether one side is higher than the other. It is difficult to obtain symmetrical images from patients with spinal deformity like normal patients, so they often cannot be accurately diagnosed (Heo et al., 2009; Moon et al., 2009). 3D-DESS can display intervertebral discs and nerve roots at the same time independent of the influence of soft tissues, which makes it easy to diagnose disc herniation in patients with spinal deformity. In terms of this advantage, 3D-DESS is remarkably superior to CT scan. As shown in Figure 4, the interpretation of CT scan, like MRI, was achieved by analyzing continuous cross-sectional images, and it was also affected by deformity, which brought difficulties to accurate diagnosis.

**FIGURE 5:**
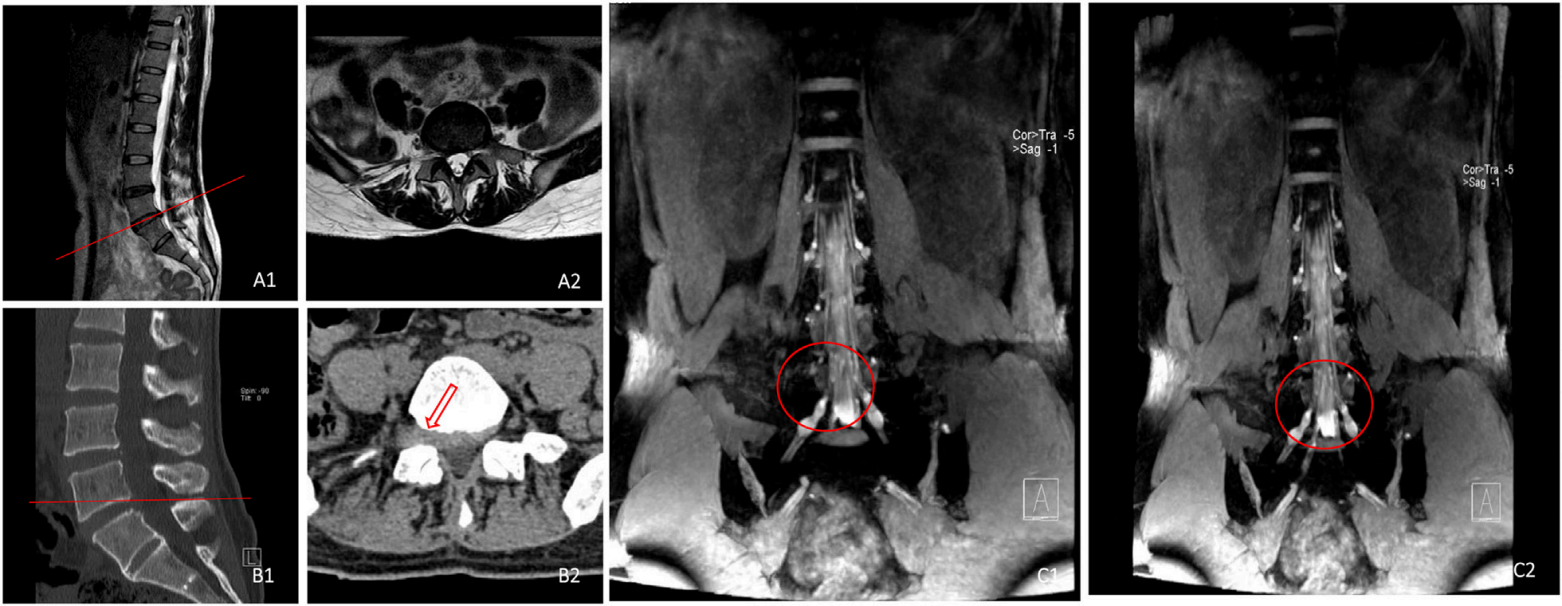
Sagittal **(A1)** and cross-sectional **(A2)** MR images indicated that the right side of L4/L5 intervertebral disc protruded, Sagittal **(B1)** and cross-sectional **(B2)** CT images displayed that the extreme lateral side of L4/L5 segment protruded greatly, and 3D-DESS image **(C1, C2)** revealed that the right L5 nerve root was compressed in the spinal canal, and the walking was interrupted. The protruded intervertebral discs on the extreme lateral side did not compress L4 nerve root, with smooth walking.

Moreover, 3D-DESS directly determines whether disc herniation involves the intervertebral plane. Through conventional MRI and CT scan, disc herniation is often diagnosed, but whether the nerve root is exactly compressed requires judgment by experienced doctors according to the clinical symptoms and signs of patients, which is also often worrying spinal surgeons. They often worry about the efficacy of operation, especially the effective alleviation of lower limb radiation pain of the patients after operation. However, the intuitiveness of 3D-DESS makes it very easy to judge the responsible spine. According to the clinical data in Figure 5, conventional MRI manifested that the right side of L4/L5 intervertebral disc protruded, while CT displayed that the extreme lateral side of L4/L5 segment protruded greatly, based on which it was impossible to judge the definite responsible intervertebral space. However, 3D-DESS revealed that the right side of L4/L5 segment was greatly protruded, the right L5 nerve root was compressed in the spinal canal, and the walk root was interrupted. Although herniation was observed in the extreme lateral intervertebral disc, it did not compress the L4 nerve root, and the entire L4 nerve root walked smoothly without intrusion. The clinical symptoms and physical examination of the patient indicated the symptoms on the right side of L5. Later, the patient’s symptoms were relieved obviously after simply treating the herniated intervertebral disc tissues in the spinal canal, which was completely consistent with the conclusion of 3D-DESS.

**FIGURE 6:**
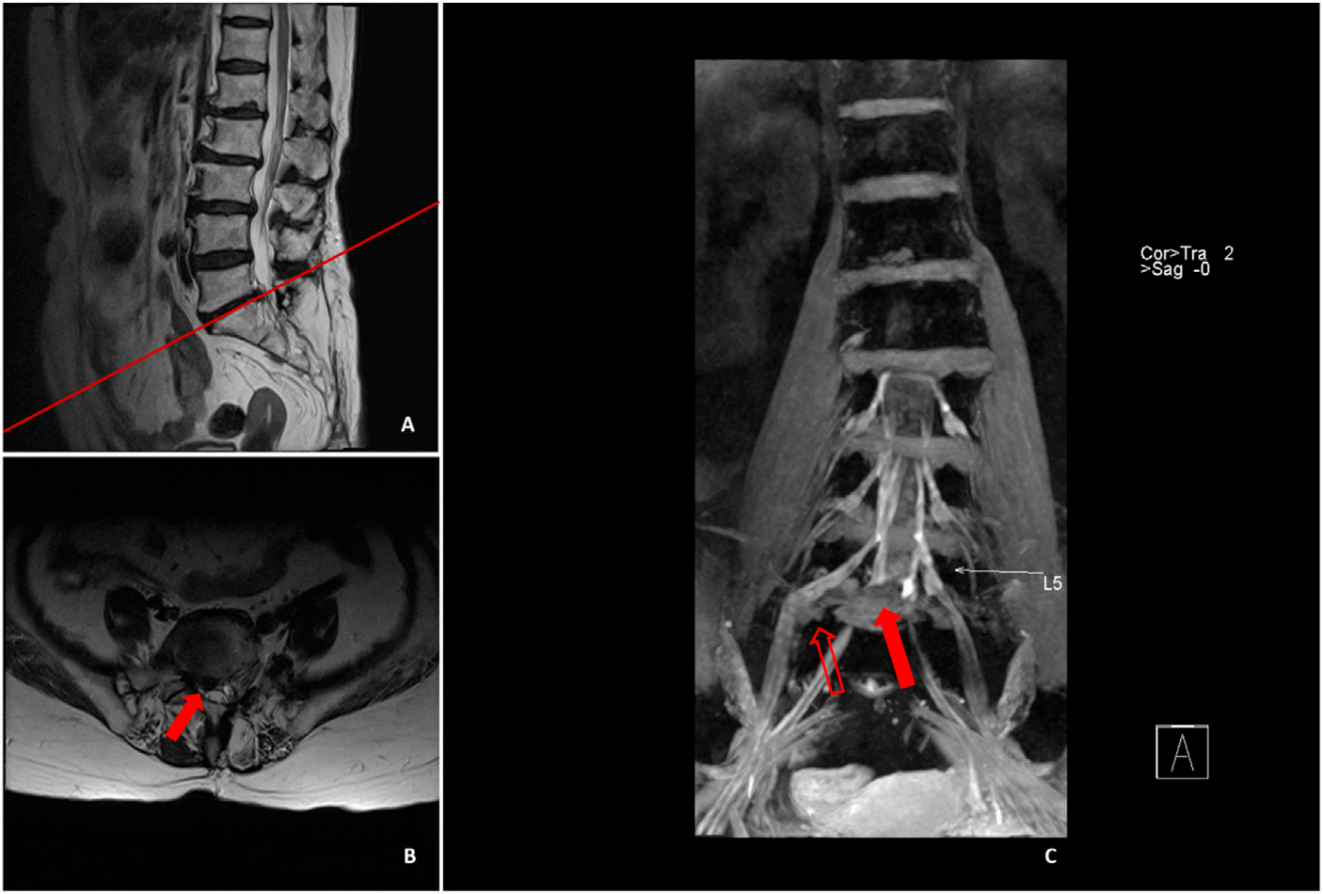
Sagittal **(A)** and cross-sectional MRI **(B)** image illustrated that L5/S1 protruded to the right side, and the preoperative physical examination manifested that the skin sensation of the dorsum and plantar of the lateral side of the right leg was decreased, with positive results in straight leg raising test. 3D-DESS **(C)** illustrated that the intervertebral disc on L5/S1 segment protruded to the right side in the spinal canal, compressing the S1 nerve root (red solid arrow), whereas the L5/S1 extreme lateral nucleus pulposus (red hollow arrow) also compressed the L5 nerve root.

In terms of another patient (Figure 6), conventional cross-sectional and cross-sectional MR images displayed that herniation occurred on the right side of L5/S1 segment, and preoperative physical examination indicated that the skin sensation on the dorsum of the lateral foot and plantar of the right leg was decreased, and the straight leg elevation test result was positive. 3D-DESS revealed that L5/S1 intervertebral disc protruded on the right side in the spinal canal, compressing S1 nerve root. In the meantime, the extreme lateral nucleus pulposus of L5/S1 segment also compressed L5 nerve root. According to the judgment of the responsible segment, the above two parts were treated during operation, and the pain of the patient was completely alleviated after operation. However, if 3D-DESS technology was not applied, the compression of the right L5 nerve root caused by the extreme lateral disc herniation of L5/S1 segment could easily be missed, resulting in partial relief of radiation pain of lower limbs after operation.

**FIGURE 7:**
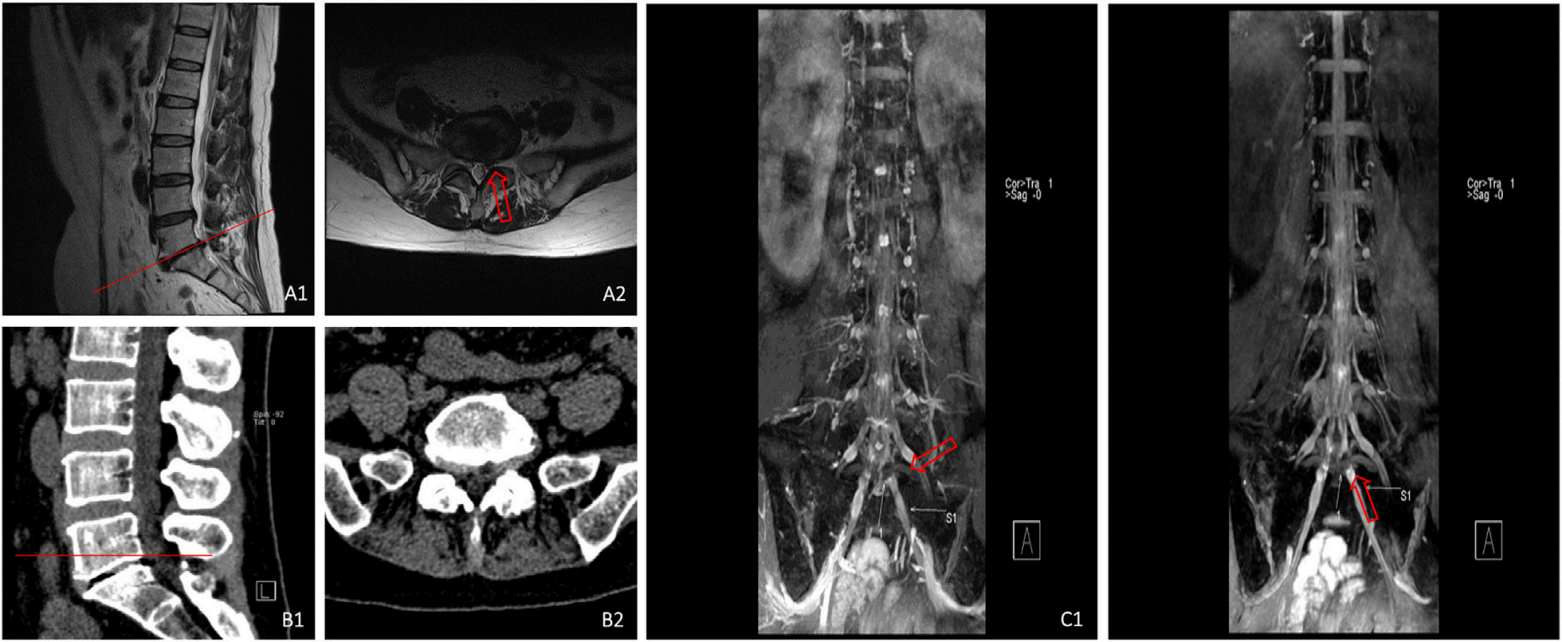
MRI **(A1,A2)** showed that the nucleus pulposus on the left side of L5/S1 segment was compressed, CT **(B1,B2)** illustrated that there was no substantial compression on the left side of L5/S1 segment, and 3D-DESS **(C1,C2)** revealed that the continuity of left S1 nerve root was interrupted with notable local compression.

In the current study, CT plain scan exhibited a high diagnostic rate for LDH-induced LBP and leg pain, which could be mutually verified with 3D-DESS sequences to further confirm the diagnosis. Nonetheless, there was an exception for a patient (Figure 7) who had typical compression symptoms of the left S1 nerve root, but could not be definitely diagnosed by CT scanas no substantial compression was detected on the left side of L5/S1 segment. Subsequent MRI manifested that the nucleus pulposus of L5/S1 segment compressed the left S1 nerve root. However, it could be observed in 3D-DESS examination that the compression to the left S1 nerve root was obvious and the continuity was interrupted, which corresponded to conventional MRI diagnosis. Intraoperative exploration suggested that local compression was not caused by nucleus pulposus, but by local gas vacuoles.

There were some limitations in this research. Firstly, this was a retrospective study with only 59 cases included, showing a small sample size. To tackle with this problem, extra imaging planes should be increased, and the sample size should be enlarged. In the future research, therefore, it is reasonable to further test the sequences with a larger sample size and additional imaging planes. Secondly, the samples of this study were definitely diagnosed with intraoperative findings as the gold standards. However, the operation was only conducted for the patients who underwent conservative treatment failure or still suffered from dysfunction with poor efficacy. The pain of a considerable number of patients could be relieved through conservative treatment, so there might be bias in the selection of samples. Thirdly, the high imaging cost of 3D-DESS examination increased the medical expenses of LDH patients who could be definitely diagnosed by conventional MRI. Therefore, the examination indications of 3D-DESS should be strictly screened, and 3D-DESS examination were generally not advised to patients.

## Conclusion

To sum up, this research confirmed the diagnostic value of 3D-DESS and CT in the diagnosis of controversial LDH, exhibiting their usefulness.

## Data Availability

The original contributions presented in the study are included in the article/supplementary material, further inquiries can be directed to the corresponding author.
